# Thrombectomy versus Medical Management for Pediatric Acute Ischemic Stroke Due to Isolated M2 Occlusion: A Multicenter Cohort Study

**DOI:** 10.1002/ana.78101

**Published:** 2025-11-28

**Authors:** Peter B. Sporns, Kartik D. Bhatia, Prakash Muthusami, Carmen Parra‐Farinas, Christine K. Fox, Adam A. Dmytriw, Basile Kerleroux, Sarah Lee, Jens Fiehler, Christian Lehmann, Franja Dugar, Todd Abruzzo, Lisa Pabst, Stuart Fraser, Lisa R. Sun, Grégoire Boulouis, Tanja Burkard, Olivier Naggara, Manoelle Kossorotoff, André Kemmling, Martin Olivieri, Mesha Martinez, Marios Psychogios, Thi Dan Linh Nguyen‐Kim, Moritz Wildgruber

**Affiliations:** ^1^ Department of Radiology and Neuroradiology Stadtspital Zürich Zürich Switzerland; ^2^ Department of Neuroradiology University Hospital Basel Basel Switzerland; ^3^ Department of Diagnostic and Interventional Neuroradiology University Medical Center Hamburg‐Eppendorf Hamburg Germany; ^4^ Children's Hospital at Westmead Clinical School University of Sydney Sydney NSW Australia; ^5^ Division of Paediatric Interventional Neuroradiology Hospital for Sick Children Toronto ON Canada; ^6^ Department of Neurology and Department of Pediatrics University of California San Francisco San Francisco CA USA; ^7^ Neurointerventional and Neuroanalytics Collaboration (NAN‐C), School of Medicine Toronto Metropolitan University Toronto ON Canada; ^8^ Department of Neuroradiology APHM La Timone Marseille France; ^9^ Department of Radiology Centre Hospitalier de Bastia Bastia France; ^10^ Division of Child Neurology, Department of Neurology Stanford University Stanford CA USA; ^11^ Department of Neurosurgery Barrow Neurological Institute Phoenix AZ USA; ^12^ Department of Radiology Phoenix Children's Hospital Phoenix AZ USA; ^13^ Division of Pediatric Neurology, Department of Pediatrics University of Utah School of Medicine Salt Lake UT USA; ^14^ Division of Child and Adolescent Neurology, Department of Pediatrics The University of Texas McGovern Medical School Houston TX USA; ^15^ Department of Neurology Johns Hopkins School of Medicine Baltimore MD USA; ^16^ Diagnostic and Interventional Neuroradiology DepartmentCIC‐IT 1415 CHRU de Tours Université de Tours, INSERM, Imaging Brain and Neuropsychiatry iBraiN U1253 Tours France; ^17^ French Center for Pediatric Stroke Paris France; ^18^ Department of Radiology LMU University Hospital, LMU Munich Munich Germany; ^19^ Pediatric Radiology Department, Necker Enfants Malades (NEM) INSERM UMR1266, Sainte‐Anne Paris France; ^20^ Department of Pediatric Neurology French Center for Pediatric Stroke, NEM Paris France; ^21^ Department of Neuroradiology University Hospital Marburg Marburg Germany; ^22^ Pediatric Thrombosis and Hemostasis Unit Dr. von Hauner Children's Hospital, LMU Munich Munich Germany; ^23^ Department of Radiology, Texas Children's Hospital Baylor College of Medicine Austin TX USA

## Abstract

**Objective:**

Endovascular thrombectomy (EVT) is increasingly used for pediatric large vessel occlusion (LVO) stroke, however, its role in isolated M2 occlusions remains underexplored. This study compared clinical outcomes in children with isolated M2 occlusion treated with EVT versus best medical therapy (BMT).

**Methods:**

This multicenter cohort study pooled data from 4 pediatric stroke registries (Save ChildS, KidClot, Pediatric LVO Study, and Save ChildS Pro). Children ages 28 days–17 years with isolated M2 occlusion presenting within 24 hours of last seen well were included. Primary outcome was the pediatric modified Rankin scale (ped‐mRS) at 3 to 6 months. Secondary outcomes included changes in Pediatric National Institutes of Health Stroke Scale (PedNIHSS), Pediatric Stroke Outcome Measure (PSOM), and safety endpoints.

**Results:**

Forty patients were included, of whom 20 were treated with EVT (median age, 12 years; interquartile range [IQR], 6–15; 40% female) and 20 with BMT only (10 years; IQR, 5–14; 50% female). Baseline demographics were similar. EVT patients showed superior outcomes: median ped‐mRS at 3 to 6 months was 1 versus 2 (*p* = 0.015). EVT resulted in greater PedNIHSS reduction from admission to day 7 (−9 vs −1, *p* < 0.001) and lower PSOM at 3 to 6 months (0.5 vs 2.5, *p* = 0.009). This benefit persisted at 24 months with a median ped‐mRS of 1 (IQR, 0–2) in the EVT group and 2 (IQR, 1–3) in the BMT group (*p* = 0.012). One symptomatic intracranial hemorrhage occurred in the BMT group, and no deaths or access‐site complications were reported.

**Interpretation:**

In children with isolated M2 occlusion, EVT was associated with better functional outcomes and neurological recovery than medical therapy alone, with an acceptable safety profile. ANN NEUROL 2026;99:684–691

Arterial ischemic stroke (AIS) affects 1.3 to 1.6 per 100,000 children annually in developed countries, with significant long‐term morbidity and mortality.^1^, ^2^, ^3^ Unlike adults, pediatric stroke is rarely caused by atherosclerosis, and systematic research is limited by recruitment challenges, as seen in the early termination of the TIPS trial.^4^

In recent years, endovascular thrombectomy (EVT) has become standard of care for adult large vessel occlusion (LVO) stroke.^5^, ^6^ Although retrospective studies like Save ChildS^7^ and Kid Clot^8^ supported EVT's safety and effectiveness in children, they were limited by the lack of control groups. The prospective multicenter Save ChildS Pro registry recently provided evidence for the safety of EVT for pediatric LVO stroke and showed an association with better neurological outcomes in children with LVO stroke who received EVT compared to those with best medical treatment (BMT) alone.^9^

Although adult trials show that EVT can benefit patients with isolated occlusions of the M2‐segment of the middle cerebral artery (MCA)^10^—albeit with smaller effect sizes than for LVOs—pediatric data are sparse. The largest series reported 6 children and focused on technical aspects and factors relevant to decision making, but systematic conclusions could not be drawn.^11^

To address this evidence gap, we undertook a pooled analysis comparing outcomes between EVT and BMT in pediatric patients with isolated M2 occlusions, using harmonized data from the 4 largest available multicenter cohort studies. We hypothesized that EVT would be associated with better functional outcomes and greater neurological improvement.

## Methods

### Study Design and Participants

We conducted a secondary analysis of de‐identified data pooled from 4 previously published multicenter pediatric stroke studies: (Save ChildS,^7^ Kid Clot,^8^ the Pediatric LVO Stroke Study,^12^ and Save ChildS Pro^9^, ^13^) (Table S1). Inclusion criteria were age 28 days to 17 years at stroke onset, acute ischemic stroke because of isolated M2 occlusion (imaging assessment partly performed by local interdisciplinary stroke team), and clinical presentation within 24 hours since last seen well (LSW). Patients with tandem occlusions or proximal ICA/M1 involvement were excluded.

All patients with M2 occlusions were stratified into 2 groups: those who underwent EVT (n = 20) and those who received BMT alone (n = 20).

Save ChildS Pro was approved by the ethics committee of the University of Münster (Münster, Germany; 2019‐677‐f‐S), in accordance with the Declaration of Helsinki, with waiver for informed consent. Institutional review board approvals and data‐sharing agreements were in place for each contributing registry.

### Outcomes

The primary outcome was the pediatric modified Rankin scale (ped‐mRS) score at 3 to 6 months after stroke onset.

Secondary outcomes included difference in Pediatric National Institutes of Health Stroke Scale (PedNIHSS) at 7 days vs admission, Pediatric Stroke Outcome Measure (PSOM) at discharge, PSOM at 3 to 6 months, ped‐mRS at 24 months (interquartile range [IQR]), rate of successful recanalisation (mTICI 2b or better) in the EVT group, rate of symptomatic intracranial hemorrhage (sICH), access site complications, and the rate of intra‐procedural vasospasm in the EVT group.

### Statistical Analysis

Continuous variables were compared using the *t* test or Mann–Whitney *U*‐test as appropriate. Categorical variables were compared using chi‐squared or Fisher's exact tests. Ordinal data (eg, ped‐mRS, PSOM) were analyzed using ordinal logistic regression or non‐parametric methods. Propensity score matching was performed adjusted for the variables sex, age, PedNIHSS at admission, and Alberta Stroke Program Early Computed Tomography Score (ASPECTS) at admission. Fourteen patients from each treatment group were matched 1:1 with an absolute difference between propensity scores of 0.2 1 adjusting for age, sex, admission PedNIHSS, and ASPECTS. The statistical analysis was carried out using SAS version 9.4 (SAS Institute, Cary, NC). A *p*‐value <0.05 was considered statistically significant.

## Results

A total of 40 pediatric patients with isolated M2 occlusion were included in the study, with 20 receiving EVT and 20 treated with best medical therapy BMT. Baseline demographics and clinical characteristics were similar between groups, including age, sex, and stroke severity at presentation. Median age was 12 years in the EVT group and 10 years in the BMT group. The EVT group had a shorter median time from symptom onset to hospital admission (86 minutes vs 192 minutes, *p* = 0.022) and had higher baseline ASPECTS scores (9 vs 7, *p* = 0.036) (Table 1).

**TABLE 1:** Demographic and Clinical Characteristics of Participants at Baseline and Treatment

	Endovascular thrombectomy (N = 20)	Best medical treatment (N = 20)	*p*
Median age (IQR), yr	12 (6–15)	10 (5–14)	0.278
Sex			0.525
F	8 (40%)	10 (50%)	
M	12 (60%)	10 (50%)	
Median PedNIHSS score at hospital arrival (IQR)	11 (8–18)	8 (4–13)	0.069
Median onset to admission (IQR), hours	1.4 (0.7–2.0)	3.2 (1.9–7.0)	0.005
Median onset to recanalization (IQR), hours	7.0 (3.9–8.0)	–	–
Median ASPECTS baseline (IQR)	9 (8–10)	7 (6–9)	0.045
Median interval between symptom onset and admission for known onset strokes (IQR), hours	2.2 (0.9–4.6)	3.8 (1.3–7.1)	0.046
Median interval between symptom onset and first recanalization pass (IQR), hours	5.7 (4.0–8.4)	–	–
Anesthesia performed (%)			
Conscious sedation or none	15 (13)	–	–
General anesthesia	102 (87)	–	–
Type of device used for thrombectomy, n (%)			
Aspiration catheter alone	31 (26)	–	–
Stent retriever alone	72 (62)	–	–
Both aspiration catheter and stent retriever	14 (12)	–	–
Median attempts for thrombectomy (IQR), n	2 (1–3)	–	–
Intravenous tPA, n (%)	6 (30)	2 (10)	0.235
Etiology according to CASCADE classification			0.163
Small vessel arteriopathy	0 (0)	0 (0)	
2 Focal cerebral arteriopathy	4 (20)	7 (35)	
3 Bilateral cerebral arteriopathy	0 (0)	0 (0)	
4 Aortic/cervical arteriopathy	0 (0)	2 (10)	
5 Cardioembolic	10 (50)	4 (20)	
6 Other	6 (30)	6 (30)	
7 Multi‐factorial	0 (0)	1 (5)	

Data are presented as total numbers n and percentages (%) unless specified. Scores on the PedNIHSS range from 0–42, with higher scores indicating greater neurological deficit. ASPECTS values range from 0–10, with lower values indicating larger infarction.

ASPECTS = Alberta Stroke Program Early Computed Tomography Score; F = female; IQR = interquartile range; M = male; PedNIHSS = Pediatric National Institutes of Health Stroke Scale; tPA = tissue plasminogen activator.

**FIGURE 1:**
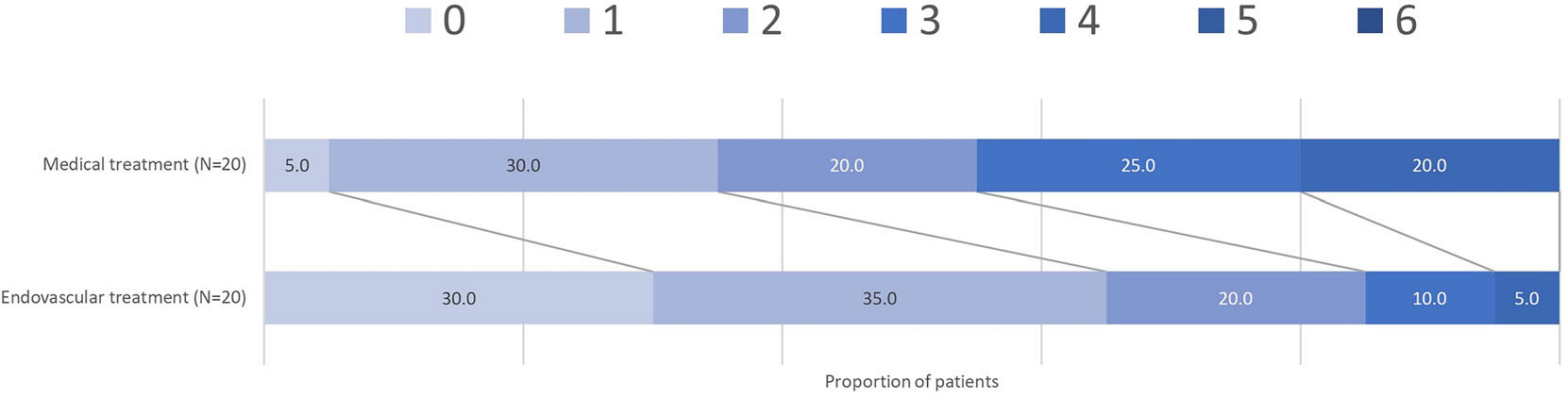
Distribution of modified Rankin scale scores at 3–6 months following stroke onset. [Color figure can be viewed at www.annalsofneurology.org]

At 3 to 6 months, children treated with EVT had better functional outcomes. The median ped‐mRS score was 1 (IQR, 0–2) in the EVT group compared to 2 (IQR, 1–3) in the BMT group (*p* = 0.015). This benefit persisted at 24 months: at that time point the median ped‐mRS was 1 (IQR, 0–2) in the EVT group and 2 (IQR, 1–3) in the BMT group (*p* = 0.012) (Table 2, Fig 1).

**TABLE 2:** Clinical and Angiographic Outcomes

	Endovascular thrombectomy (N = 20)	Best medical treatment (N = 20)	*p*
Median score on modified Rankin scale at 3–6 mo (IQR)	1 (0–2)	2 (1–3)	0.015
Median score on Pediatric Stroke Outcome Measure at 3–6 mo (IQR)	0.5 (0–2)	2.5 (1–4)	0.009
Median difference of Pediatric NIHSS at 7 days vs admission (IQR)	−9 (−10 to −6)	−1 (−3 to 0)	<0.001
Median score on Pediatric Stroke Outcome Measure at discharge (IQR)	1 (1–2)	3 (1.5–3.5)	0.033
Median score on modified Rankin scale at 24 mo (IQR)	1 (0–2)	2 (1–3)	0.012
Successful recanalisation (mTICI 2b or better)	19 (95%)	–	–
Complete recanalisation (mTICI 3)	6 (30%)	–	–
Median ASPECTS follow‐up (IQR)	8 (7–9)	6 (4–8)	0.063

The rate of successful restoration of flow of the occluded target arterial vessel, defined as mTICI 2b or better, was reported descriptively for the group that received thrombectomy with 95% CIs based on the Wald method. Scores on the modified Rankin scale range from 0–6, with higher scores indicating greater disability. Successful reperfusion was defined as grade 2b–3 on the modified thrombectomy in the cerebral ischemia system ranging from 0–3, with higher grades indicating increased reperfusion; grade 2b indicates reperfusion of ≥50% of the occluded cerebral artery territory; and grade 3 indicates reperfusion of 100% of the occluded cerebral artery territory at the end of the thrombectomy procedure.

ASPECTS = Alberta Stroke Program Early Computed Tomography Score; CI = confidence interval; IQR = interquartile range; NIHSS = National Institutes of Health Stroke Scale.

**FIGURE 2:**
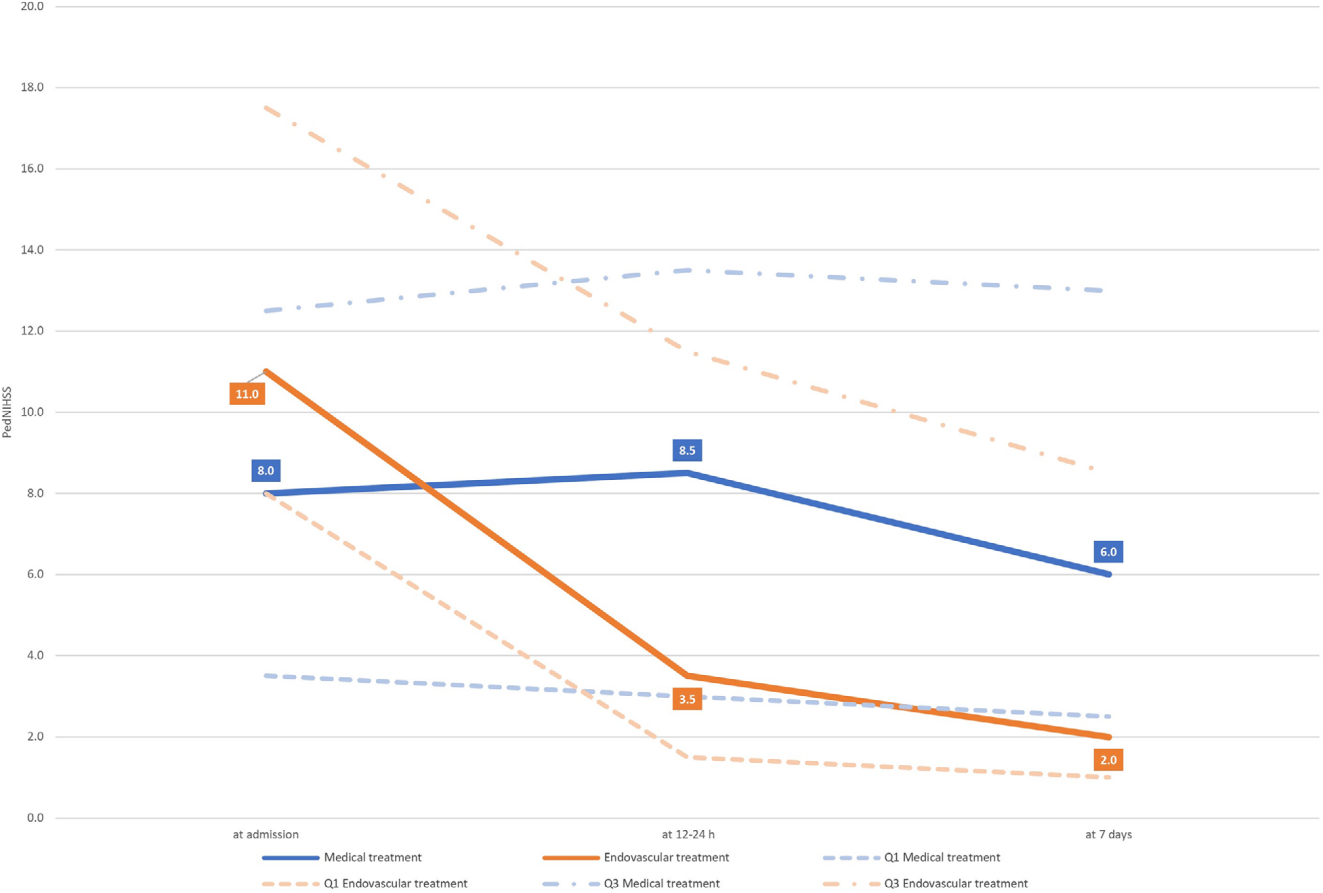
Course of Pediatric National Institutes of Health Stroke Scale from admission to day 7. [Color figure can be viewed at www.annalsofneurology.org]

**FIGURE 3:**
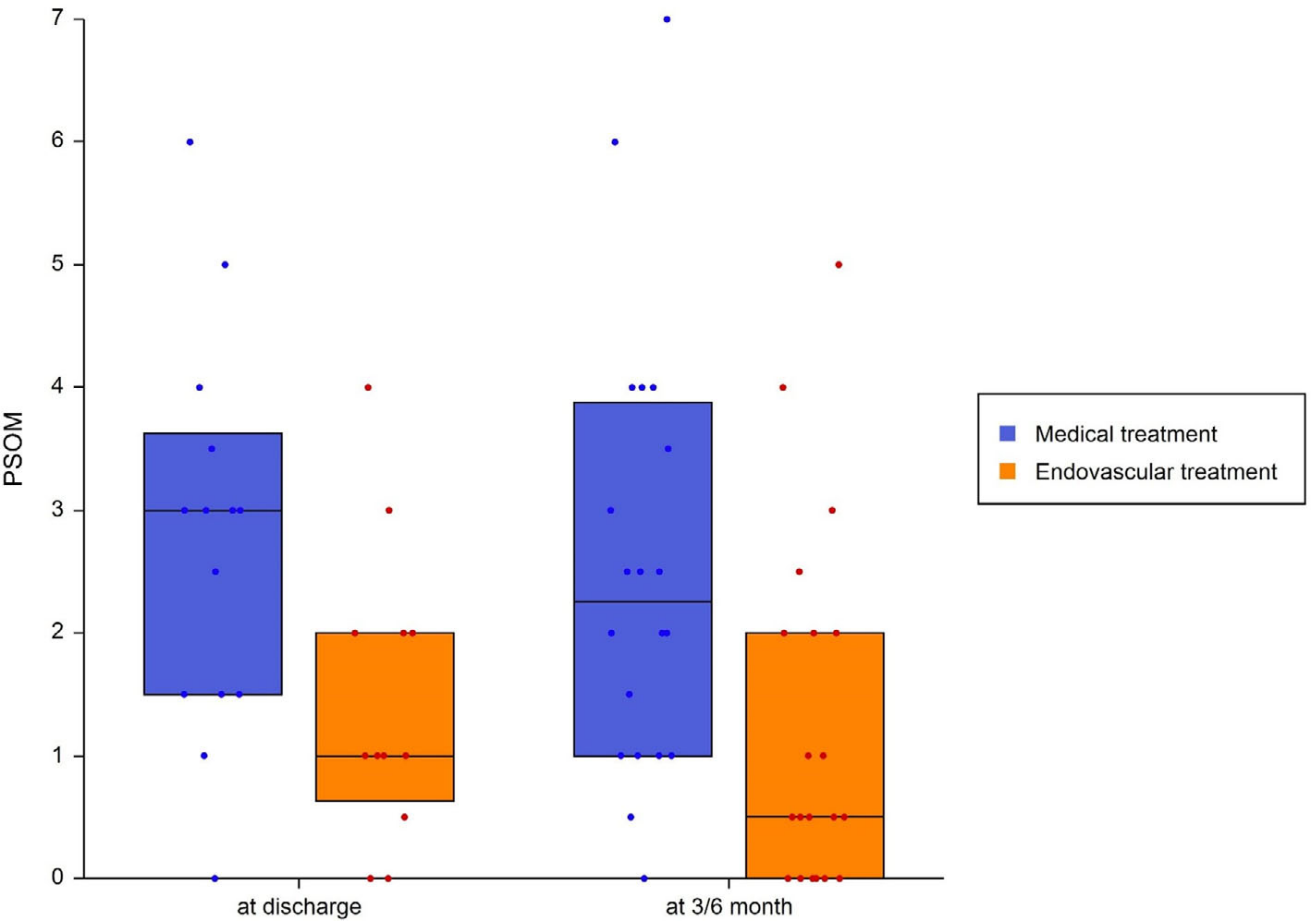
PSOM at discharge and 3–6 months post‐stroke. Values in boxplots show median and interquartile range in both the EVT and BMT group at discharge and at follow‐up. Dots are single patients in total range. BMT = best medical therapy; EVT = endovascular thrombectomy; PSOM = Pediatric Stroke Outcome Measure. [Color figure can be viewed at www.annalsofneurology.org]

Neurological improvement within the first week after stroke was also greater in the EVT group. The median reduction in PedNIHSS from admission to day 7 was −9 (IQR, −10 to −6) for EVT compared with −1 (IQR, −3 to 0) for BMT (*p* < 0.001) (Fig 2). At discharge, the PSOM was lower in EVT patients (median, 1; IQR, 1–2) compared to BMT (median, 3; IQR, 1.5–3.5; *p* = 0.033). At 3 to 6 months, median PSOM scores remained better in the EVT group (0.5; IQR, 0–2) versus BMT (2.5; IQR, 1–4; *p* = 0.009) (Fig 3).

Successful recanalization (defined as mTICI 2b or better) was achieved in 95% of EVT patients. Complete recanalization (mTICI 3) was achieved in 30%. Safety outcomes were similar across groups. One symptomatic intracranial hemorrhage occurred in the BMT group (5%) and none in the EVT group. There were no deaths or access‐site complications reported. Transient intra‐procedural vasospasm occurred in 3 patients (15%) undergoing EVT (Table 3).

**TABLE 3:** Safety Outcomes

	Endovascular thrombectomy (N = 20)	Medical treatment (N = 20)	*p*
Death (modified Rankin scale 6) at 3–6 mo	0 (0%)	0 (0%)	–
Symptomatic intracranial hemorrhage	0 (0%)	1 (5%)	–
Transient intra‐procedural vasospasm	3 (15%)	–	–

Data are n (%) unless specified. Symptomatic intracranial hemorrhage was defined as an increase in the PedNIHSS score of ≥4 points with presence of parenchymal hemorrhage.

PedNIHSS = Pediatric National Institutes of Health Stroke Scale.

A propensity score–matched analysis of 14 EVT and 14 BMT patients confirmed these findings. In this matched cohort, EVT remained associated with significantly better functional outcomes at 3 to 6 months (median ped‐mRS, 1 vs 3; *p* = 0.029), better PSOM scores at 3 to 6 months (median, 0.5 vs 2.5; *p* = 0.013), greater early neurological improvement (median PedNIHSS reduction, −8 vs 0; *p* = 0.014), and better long‐term outcomes at 24 months (median ped‐mRS, 1 vs 3; *p* = 0.032) (Table 4).

**TABLE 4:** Propensity Score Matched Analysis

	Endovascular thrombectomy (N = 14)	Medical treatment (N = 14)	*p*
Median score on modified Rankin scale at 3–6 mo (IQR)	1 (0–2)	3 (1–4)	0.029
Median score on Pediatric Stroke Outcome Measure at 3–6 mo (IQR)	0.5 (0–2)	2.5 (1.5–4)	0.013
Median difference of Pediatric NIHSS at 7 days vs admission (IQR)	−8 (−10 to −4)	0 (−3–0)	0.014
Median score on modified Rankin scale at 24 mo (IQR)	1 (0–2)	3 (1–3)	0.032

Data are n (%) unless specified. Scores on the PedNIHSS range from 0–42, with higher scores indicating greater neurological deficit. Scores on the modified Rankin scale range from 0–6, with higher scores indicating greater disability. Scores on the Pediatric Stroke Outcome Measure range from 0–10 in 0.5 steps, with higher scores indicating greater disability.

IQR = interquartile range; PedNIHSS = Pediatric National Institutes Of Health Stroke Scale.

## Discussion

In this multicenter cohort study of children with isolated M2 occlusion, EVT was associated with better functional outcomes compared to BMT alone, despite a trend towards higher PedNIHSS at presentation in the EVT group. The group of children with EVT had higher rates of favorable outcomes (ped‐mRS, PSOM), greater early neurologic improvement, and no increase in symptomatic intracranial hemorrhage or procedural complications. These findings support the expanding role of EVT in carefully selected children with stroke and an isolated M2 occlusion.

Although results from of a meta‐analysis from the HERMES collaboration have suggested benefit of EVT in proximal M2 occlusions with modest effect sizes,^10^ recent randomized controlled trials including distal intracranial occlusions^14^, ^15^ did not show better functional outcomes after EVT compared to BMT alone. Nonetheless our results suggest that for M2 occlusions there is a robust treatment effect of EVT in children, potentially because of greater neuroplasticity, better collateral circulation,^16^, ^17^ and fewer comorbidities.^1^ Notably, successful recanalization (mTICI 2b–3) was achieved in 95% of EVT cases, which may also be attributed to the lower prevalence of intracranial atherosclerosis and the lower rate of difficult vascular access because of vessel tortuosity in children.^18^ Additionally, focal cerebral arteriopathy, a frequent etiology of stroke in children and a factor possibly complicating EVT, is usually confined to the intracranial carotid artery and the M1‐segment.^19^, ^20^ Importantly, the EVT group showed a meaningful reduction in NIHSS at 24 hours, indicating early neurologic benefit.

Although baseline demographics were generally well balanced between treatment groups, some clinically relevant differences were observed. The EVT group had a higher median PedNIHSS score at presentation (11 vs 8), suggesting more severe initial neurological deficits, although this difference did not reach statistical significance (*p* = 0.081). Additionally, patients in the EVT group presented earlier, with significantly shorter onset‐to‐admission times (86 vs 192 minutes, *p* = 0.022), and had higher baseline ASPECTS scores (9 vs 7, *p* = 0.036), indicating smaller initial infarcts.

To mitigate these baseline imbalances and reduce potential confounding, we performed a propensity score matched comparison, which confirmed the superiority of EVT across all primary and secondary outcome measures, supporting the robustness of our findings and suggesting that the observed treatment effect is unlikely to be fully explained by baseline differences alone.

Regarding safety, there was no difference in the rate of symptomatic intracranial hemorrhage between groups, suggesting EVT in M2 occlusion carries an acceptable safety profile.

Overall, our findings suggest that children with acute M2 occlusion should not be automatically excluded from consideration for EVT. In a setting where conducting randomized trials is unlikely feasible because of recruitment challenges for M2 occlusions and the difficulty of randomizing children for a treatment that has shown a strong effect in adults for LVOs, this study provides practical evidence supporting individualized decisions in pediatric stroke management, particularly when access to pediatric stroke expertise and endovascular teams are available. It also emphasizes the importance for international collaborations for the study of rare diseases, which may be used to inform future guidelines.

This study is limited by its retrospective, non‐randomized design and relatively small sample size. Matching attempted to reduce bias, but unmeasured confounders may still influence outcomes. Imaging protocols were heterogeneous and occlusion location and ASPECTS were not centrally adjudicated. Included cohorts were heterogeneous, which may also be a strength by expanding geographic representation and increasing generalizability. Especially, the patients in the EVT group presented earlier and had higher baseline ASPECT scores compared to the BMT group. Last, this presents by far the largest available pediatric M2 occlusion dataset, pooling data from 4 major registries.

## Conclusion

This pooled analysis suggests that EVT for pediatric stroke because of isolated M2 occlusion may lead to improved functional outcomes compared with medical therapy alone. Therefore, EVT should be considered in carefully selected pediatric patients with M2 occlusion. Further prospective data and international consensus are needed to guide standardized practice.

## AUTHOR CONTRIBUTIONS

P.B.S., and M.W. contributed to the conception and design of the study; P.B.S., K.D.B., P.M., C.P.F., C.K.F., A.A.D., B.K., S.L., J.F., C.L., F.D., T.A., L.P., S.F., L.R.S., G.B., T.B., O.N., M.K., A.K., M.O., M.M., M.P., T.D.L.N.K., and M.W. contributed to the acquisition and analysis of data; P.B.S., T.B., and M.W. contributed to drafting the text or preparing the figures. [Correction added on 25 February 2026, after first online publication: Author contribution text has been revised in this version.]

## Potential Conflicts of Interest

Nothing to report.

## Supporting information


**Table S1.** Major studies on hyperacute recanalization in children.

## Data Availability

The data analyzed for the current study are available from the corresponding author on reasonable request from the moment of publication and with a signed data access agreement.
