# Supplementary material for: Thrombectomy versus Medical Management for Pediatric Acute Ischemic Stroke Due to Isolated M2 Occlusion: A Multicenter Cohort Study
Source: Ann Neurol. 2025 Nov 28;99(3):684–91. doi: 10.1002/ana.78101 (PMC12954143; doi:10.1002/ana.78101)
Supplement: Supplementary file 1 — Table S1. Major studies on hyperacute recanalization in children. [file ANA-99-684-s001.docx]

**Supplement**

**Supplemental Table 1**

Major studies on hyperacute recanalization in children

| Study | Enrollment period | Centers | | Inclusion criteria | Imaging review | Age / Sex | N /  % EVT | Notes |
| --- | --- | --- | --- | --- | --- | --- | --- | --- |
| Save ChildS (JAMA Neurol, 2020) | Jan 2000 – Dec 2018 | | 27 stroke centers, Europe + USA | Children <18 yrs with AIS and LVO who underwent EVT | Imaging and outcome review at sites; no central review | N=73; median age 11.3 y; 51% male | 73/73 (100%) | Largest early multicenter EVT cohort; retrospective, safety & feasibility study |
| KidClot (JAMA Netw Open, 2022) | Jan 2015 – May 2018 | | 30 centers across France | Children 28 days–18 yrs with AIS who received IVT and/or EVT | Central blinded review by 3 neuroradiologists | N=68; median age 11 y; 65% male | 40/68 (59%) | Nationwide cohort; assessed IVT, EVT, or both; centralized adjudication |
| Paediatric LVO Study (JAMA Neurol, 2023) | Jan 2011 – Apr 2022 | | 5 centers, Australia + Canada | 1 mo–<18 yrs with acute LVO stroke; neonates excluded | Blinded neuroradiologist in each country reviewed occlusion; site-based imaging | N=52; mean age 10.3 y; 60% male | 26/52 (50%) | Matched case-control study; EVT vs medical management; improved outcomes with EVT |
| Save ChildS Pro (Lancet Child Adolesc Health, 2024) | Jan 2020 – Aug 2023 | | 45 centers, 12 countries (Europe, Americas, Asia, Australia) | 28 days–18 yrs; AIS with LVO/MVO; neonates excluded | Local site review; outcomes assessed locally, centrally checked for plausibility | N=208; median age EVT 11 y vs BMT 6 y; 55% male | 117/208 (56%) | Prospective multinational registry; compared EVT vs best medical therapy |
